# The Conundrum of Dystonia in Essential Tremor Patients: How does One Classify these Cases?

**DOI:** 10.5334/tohm.690

**Published:** 2022-05-13

**Authors:** Wenqin Du, Peter G. Bain, Giovanni Defazio, Joseph Jankovic, Christine Y. Kim, E. K. Tan, Marie Vidailhet, Elan D. Louis

**Affiliations:** 1 Department of Neurology, University of Texas Southwestern Medical Center, Dallas, Texas, USA; 2 Department of Neurosciences, Division of Brain Sciences, Imperial College London, London, UK; 3 Department of Medical Sciences and Public Health, University of Cagliari, Monserrato, Italy; 4 Parkinson’s Disease Center and Movement Disorders Clinic, Department of Neurology, Baylor College of Medicine, Houston, Texas, USA; 5 Department of Neurology, Columbia University Medical Center, New York City, New York, USA; 6 Department of Neurology, National Neuroscience Institute, Singapore General Hospital, Singapore; 7 Sorbonne Université, Institut du Cerveau – Paris Brain Institute – ICM, Inserm, CNRS, AP-HP, Hôpital Salpetriere, DMU Neuroscience 6, Paris, France

**Keywords:** essential tremor, dystonia, diagnosis, classification

## Abstract

**Background:**

The relationship between essential tremor (ET) and dystonia has been long debated and the boundaries between these disorders remain unclear. Here, we highlight the diagnostic uncertainty that can arise when observing dystonic postures in patients who have received ET diagnoses.

**Methods:**

An international panel of seven movement disorders neurologists from five countries reviewed the clinical history and videotaped neurological examinations of five individuals diagnosed with ET who also had various features of dystonia on neurological examination. Experts were instructed to assign diagnoses and provide their rationale for diagnostic assignments.

**Results:**

The five cases each exhibited a variety of abnormal postures. These were observed by all experts, and interpreted as dystonic postures by six experts. According to six of seven experts, all five cases had ET. One expert classified all cases as dystonic tremor rather than ET. One case had cervical dystonia, and five of seven experts assigned dual diagnoses of ET and dystonia in that case. The assignment of dystonia diagnoses was variable among the other four cases, with two to three experts assigning this diagnosis in each case, underscoring differences in diagnostic interpretation of dystonic postures on examination.

**Conclusions:**

This study draws attention to some of the differences between experts in assigning diagnoses of ET or dystonia to individuals with ET and abnormal postures. The goal here was not necessarily to build consensus, but to raise issues, highlight areas of uncertainty, and identify areas of common vs. differentiated thought. Several questions for additional research were also raised.

## Introduction

The 1998 consensus statement of the Movement Disorder Society (MDS) on Tremor defined essential tremor (ET) as a bilateral, symmetric, postural or kinetic tremor in the absence of abnormal neurologic signs, including dystonia [1]. Yet, in daily practice as well as peer-reviewed publications, dystonic postures are often noted in patients diagnosed as ET, with prevalence of such postures ranging from 0% to 47% [2,3,4]. In one series of 300 ET patients, 25.0% had dystonia distributed as follows: cervical (69.3%), limb (41.3%), laryngeal (9.3%), and blepharospasm (4.0%) [5]. Similarly, tremor is often noted in patients diagnosed with dystonia, with some series reporting a prevalence as high as 87% [6,7,8,9]. These and other observations have sparked dialogue and debate about the relationship between the two disease entities, ET and dystonia [10]. One opinion is that ET and dystonia are different diseases that can coexist in one individual [3,11]. The other opinion is that dystonic postures may be a feature seen in patients with ET [12]. It is also well recognized that patients with dystonia may have various types of postural and kinetic tremors that resemble ET. The debate is as much about uncertainties in the diagnosis of ET as it is about the uncertainties in diagnosing dystonia.

Currently, two types of dystonic tremor syndromes are recognized: 1) dystonic tremor (tremor in a body part affected by dystonia), and 2) tremor associated with dystonia (tremor that occurs in a body part not affected by dystonia, but in a patient who has dystonia elsewhere) [13]. In the 2018 consensus statement, the International Parkinson and MDS proposed a new term, “ET-plus”, to describe ET with additional neurological signs of uncertain significance such as questionable dystonic posturing. It further noted that ET-plus does not include other syndromes such as dystonic tremor and task-specific tremor [13]. Even with the new proposed terminology, there remain significant areas of uncertainty and high potential for differing diagnoses in the clinical setting. This presents a daily challenge for experts in the field [14].

Here, we present five cases diagnosed with ET, as further described below. Each was observed by the senior author (E.D.L.) to exhibit dystonic postures on examination, with a range of such dystonic features across these individuals. An international panel of movement disorders experts was invited to review the clinical features of these cases and provide educated opinions about the clinical phenomenology and the diagnostic implications of those.

## Methods

### Introduction

Two ET study databases, Risk Factors Underlying Essential Tremor (RULET) and Cognitive Study of Essential Tremor (COGNET), were queried to identify ET cases with signs of dystonia. We utilized these two databases because of the richness and detail of the data on medical history and neurological examination and the deep phenotyping provided by a senior movement disorders neurologist (E.D.L). Ultimately, five cases were selected by the senior author to showcase differing clinical presentations and severities of abnormal postures in patients diagnosed as ET (step 1, below). More specifically, these embodied ET cases with a range of different locations (e.g., limb, neck) as well as various types and levels of severity of abnormal movements. These cases were reviewed by an international panel of seven movement disorders neurologists who each assigned diagnoses (step 2, below). Their responses were organized in a table and compared (step 3, below). RULET and COGNET was approved by the institutional review board of the University of Texas Southwestern Medical Center. Informed consent was obtained from all participants and permission was given to videotape a neurological examination and use this for educational and publication purposes.

Here we describe each ET study database.

### Rulet

RULET is a cross-sectional, observational study of environmental factors associated with ET (2000 – present) [15]. ET cases underwent a videotaped neurological examination. This examination was reviewed by a movement disorders neurologist with longstanding expertise in tremor (E.D.L.) and an ET diagnosis was assigned using the Washington Heights-Inwood Genetic Study of Essential Tremor (WHIGET) criteria [16]. Any abnormal postures were also noted. The study collected extensive medical history (tremor history, family history, and medications).

### Cognet

COGNET is a longitudinal, observational study to characterize patterns of cognitive functioning among individuals with ET (2014 – present) [17]. ET cases underwent a cognitive and videotaped neurological examination every 18 months. For full details of methods, including ET assigned diagnoses using the WHIGET criteria, see [17].

As noted above, this study involved three steps.

#### Step 1 (Selection of five individuals diagnosed as ET who also had abnormal postures on examination)

We selected ET cases who met the following inclusion criteria: (1) WHIGET diagnosis of ET, (2) longstanding action tremor (>10 years since age of tremor onset), (3) family history of ET or tremor, (4) presence of concomitant abnormal postures (i.e., possible dystonic features) on videotaped neurological examination, as noted by E.D.L., (5) adequate performance of neurological examination tasks on videotaped neurological examination. The search yielded 19 cases. We selected five cases that would showcase differing clinical presentations and severities of abnormal postures. Videotape segments of the following tasks were compiled for each case to show action tremor: (1) pouring water, (2) drinking from cup, (3) finger-nose-finger maneuver and (4) drawing an Archimedes spiral. We also added specific video clips where abnormal postures was observed by E.D.L.

#### Step 2 (History and videotaped neurological examinations of five cases reviewed by an international panel of seven movement disorders neurologists)

An international panel (five countries, three continents) of seven movement disorders neurologists (Peter G. Bain, Giovanni Defazio, Joseph Jankovic, Christine Y. Kim, E.K. Tan, Elan D. Louis, and Marie Vidailhet) with extensive expertise and peer-reviewed publication record in dystonia and/or ET were asked to review a brief synopsis of the five selected cases including data on age and gender, age of tremor onset, family history and ancestry, prescribed medications, prior tremor diagnoses, past medical history, and action tremor scores as assigned using the WHIGET Tremor Rating Scale (***Table 1***). Then they reviewed the videotaped neurological examination (***Videos 1, 2, 3, 4, 5***). The experts completed the following questions: (1) what specific dystonic features did you observe (see ***Table 2***)? (2) What diagnosis would you assign (see ***Table 3***)? Lastly, the experts were instructed to provide a brief synopsis of their rationale for each answer. The goal was to obtain a rich multiplicity of experiences and vantage points.

**Table 1:** Patient history and features of ET on videotaped neurological examination.


CASE NUMBER	1	2	3	4	5

**Age (in years)**	75	72	80	82	88

**Age of Tremor Onset (in years)**	14	35	50	25	14

**Jewish Ancestry**	Yes	Yes	Yes	No	Yes

**Family History of Tremor or ET**	Mother and sister have been diagnosed with ET	Grandmother had non-specific tremor	Father, sister, and grandmother had non-specific tremor	Father and brother have been diagnosed with ET. Grandmother is suspected to have ET as well	Mother has been diagnosed with ET

**Current Medications**	atenolol	propranolol, gabapentin, duloxetine, estrodiol/norethisterone acetate	propranolol	pindolol, primidone,	mirtazapine, levothyroxine, escitalopram

**Handedness**	Right	Right	Left	Right	Right

**Total Tremor Score (out of 36)**	25.5	27	21.5	29.5	25.5

**Postural Tremor Rating**	Dominant: 1.0	Dominant: 0.5 – 1.0	Dominant: 1.0	Dominant: 1.5	Dominant: 1.0

	Non-dominant: 1.5	Non-dominant: 0.5 – 1.0	Non-dominant: 0.5	Non-dominant: 0.5	Non-dominant: 1.5

**Pouring Water Tremor Rating**	Dominant: 2.0	Dominant: 2.0 – 3.0	Dominant: 2.0	Dominant: 3.0	Dominant: 2.0

	Non-dominant: 2.0	Non-dominant: 2.0 – 3.0	Non-dominant: 2.0	Non-dominant: 3.0	Non-dominant: 2.0

**Drinking from Cup Tremor Rating**	Dominant:3.0	Dominant: 2.0 – 3.0	Dominant: 2.0	Dominant: 3.0	Dominant: 3.0

	Non-dominant: 2.0	Non-dominant: 2.0 – 3.0	Non-dominant: 2.0	Non-dominant: 3.0	Non-dominant: 2.0

**Using Spoon with Water Tremor Rating**	Dominant: 3.0	Dominant: 3.0	Dominant: 3.0	Dominant: 4.0	Dominant: 3.0

	Non-dominant: 3.0	Non-dominant: 3.0	Non-dominant: 3.0	Non-dominant: 4.0	Non-dominant: 3.0

**Finger-Nose-Finger Tremor Rating**	Dominant: 1.5	Dominant: 1.5	Dominant: 1.0	Dominant: 2.0	Dominant: 2.0

	Non-dominant: 1.5	Non-dominant: 2.0	Non-dominant: 1.5	Non-dominant: 1.5	Non-dominant: 2.0

**Drawing an Archimedes Spiral Tremor Rating**	Dominant: 2.0	Dominant: 2.0	Dominant: 2.0	Dominant: 2.0	Dominant: 2.0

	Non-dominant:3.0	Non-dominant: 2.0	Non-dominant: 1.5	Non-dominant: 2.0	Non-dominant: 2.0


Tremor scored using the Washington Heights Genetic Study of ET Rating Scale, range = 0–3 (severe) [17].

**Video 1:** **Case 1.** On frontward arm extension, the right pinky flexes downward and there is some “spooning” (abnormal dystonic flexion of the wrist and hyperextension of the fingers) and dystonic posturing of the fingers of the left hand (extension of digits 2 and 5 as well as flexion of digit 3). In the wingbeat position, the right pinky flexes slightly but less so than described above. The head is slightly yet consistently tilted to the right. There is also a transient and subtle eyebrow tremor. At the initiation of gait, there is slight pointing of the right index finger, which lessens as he walks.

**Video 2:** **Case 2.** On frontward arm extension, there is slight flaying and extension of the 4th and 5th digits of the right hand. During the wingbeat position, the right wrist is flexed and the left pinky flexes downwards. While pouring, the left pinky extends off of the glass. While walking, there is sustained pointing on the left index finger).

**Video 3:** **Case 3.** On arm extension, there is a moderate degree of flexion of the left 3rd digit at the MCP joint. This is also present during the wing beat position.

**Video 4:** **Case 4.** On forward arm extension, there is mild hyper-extension of the right 2nd digit at the MCP joint. On the left, there is clear spooning with moderate and clear hyper-extension of the 2nd digit at the MCP joint. In the wing-beat position, the dystonia is even more evident, with mild to moderate hyper-extension of the 2nd right digit at the MCP joint, and with marked spooning on the left with hyperextension of the 2nd - 4th digits at the MCP joints and splaying of the left pinky.

**Video 5:** **Case 5.** On arm extension, left digit 2 is mildly to moderately flexed downward at the MCP joint. In the wing-beat position, the left digit 2 is again mildly flexed at the MCP joint and the pinky splays slightly outward away from the other fingers. While walking, there is subtle but consistent pointing on the right.

**Table 2:** Dystonic features observed by experts in each case.


	EXPERT 1	EXPERT 2	EXPERT 3	EXPERT 4	EXPERT 5	EXPERT 6	EXPERT 7 **

Case 1							

A. Dystonic postures of the fingers and wrist							

A.1 Extension of the left 2^nd^ and 5^th^ digit during arm extension	✓	✓*		✓	✓	✓	

A.2 Flexion of the left 3^rd^ digit during arm extension	✓	✓*		✓*	✓*		

A.3 Flexion of the right 5^th^ digit during arm extension	✓	✓*			✓		

A.4 Flexion of the right 5^th^ digit during wingbeat position	✓	✓*			✓		

A.5 Spooning of left hand during arm extension		✓*			✓		

A.6 Extension of the right 2^nd^ digit when walking		✓*		✓	✓		

B. Cervical dystonia	✓		✓	✓	✓	✓	

C. Craniofacial tremor	✓		✓		✓		

Case 2							

A. Dystonic postures of the fingers and wrist							

A.1 Extension of the right 4^th^ and 5^th^ digit during arm extension		✓*		✓	✓	✓	

A.2 Flexion of right wrist during wingbeat position	✓	✓*		✓	✓		

A.3 Flexion of left wrist during wingbeat position		✓*					

A.4 Flexion of the left 5^th^ digit during wingbeat position	✓	✓*		✓	✓	✓	

A.5 Extension of the left 5^th^ digit while pouring	✓	✓*		✓	✓	✓	

A.6 Extension of the left 2^nd^ digit when walking	✓	✓*		✓	✓		

A.7 Extension of the left 5^th^ digit when walking	✓	✓*					

Case 3							

A. Dystonic postures of the fingers							

A.1 Flexion of the left 3^rd^ digit during arm extension	✓	✓*		✓	✓	✓	

A.2 Flexion of the left 3^rd^ digit during wingbeat position	✓	✓*			✓		

Case 4							

A. Dystonic postures of the fingers and wrist							

A.1 Hyper-extension of the right 2^nd^ digit during arm extension		✓*	✓		✓		

A.2 Hyper-extension of the left 2^nd^ digit during arm extension	✓	✓*		✓	✓		

A.3 Hyper-extension of the right 2^nd^ digit during wingbeat position		✓*	✓		✓		

A.4 Hyper-extension of the left 2^nd^-4^th^ digit during wingbeat position	✓	✓*		✓	✓	✓	

A.5 Splaying of left 5^th^ digit during wingbeat position	✓	✓*	✓	✓	✓	✓	

A.6 Spooning of left hand during frontward arm extension and wingbeat position	✓	✓*		✓	✓		

A.7 Flexion of left wrist	✓	✓*					

B. Jerky tremor	✓						

Case 5							

A. Dystonic postures of the fingers							

A.1 Flexion of the left 2^nd^ digit during arm extension		✓*		✓	✓	✓	

A.2 Flexion of the left 2^nd^ digit during wingbeat position		✓*		✓	✓		

A.3 Flexion of left 3^rd^ digit	✓	✓*					

A.4 Hyper-extension of left 4^th^ digit	✓	✓*					

A.5 Splaying of left 5^th^ digit during wingbeat position		✓*			✓	✓	

A.6 Extension of the right 2^nd^ digit when walking	✓	✓*		✓	✓		


* Reported “dystonic posturing of fingers” with no further description. ** Observed many of the listed phenotypes but did not interpret them as dystonic features.

**Table 3:** Diagnosis assigned by experts in each case.


	EXPERT 1	EXPERT 2	EXPERT 3	EXPERT 4	EXPERT 5	EXPERT 6	EXPERT 7

**Case 1**	dystonic tremor	ET + dystonia	ET + dystonia	ET + dystonia	ET + dystonia	ET	ET

**Case 2**	dystonic tremor	ET + dystonia	ET	ET plus	ET	ET	ET

**Case 3**	dystonic tremor	ET + dystonia	ET	ET	ET	ET	ET

**Case 4**	dystonic tremor	ET + dystonia	ET + dystonia	ET plus	ET	ET	ET

**Case 5**	dystonic tremor	ET + dystonia	ET	ET plus	ET	ET + PD	ET


#### Step 3 (Expert responses were organized and compared)

All abnormal postures observed by the experts were enumerated (***Table 2***). Each expert’s diagnosis was also provided (***Table 3***). To assess diagnostic agreement, SPSS (version 27) was used to compute the Cohen’s weighted Kappa statistic for each pair of raters, resulting in 21 comparisons. We also present each expert’s synopsis of their rationale for the diagnostic assignment.

The approach we use embodies many of the features of qualitative research.

## Results

### Dystonic features on examination: Expert opinions

The five cases each exhibited a variety of abnormal postures of the wrist, hand or fingers. This was uniformly noted by all seven experts, and interpreted as dystonic postures by all but one, who interpreted the abnormal postures in a different light. Case 1 had abnormal neck postures on examination, noted by all experts and interpreted as a dystonic posture by six of seven experts (***Table 2***).

### Final diagnoses assigned: Expert opinions

***Table 3*** shows the final diagnoses assigned by each expert. According to six of seven experts, these cases all had ET, although one expert preferred the new term “ET-plus” as an ET designation. One expert diagnosed all cases as dystonic tremor rather than ET. Six of seven experts recognized the presence of dystonic postures on examination in each case. Case 1 had cervical dystonia and for this case, five of seven experts assigned diagnoses of dystonia. Two did not. Six of seven experts still assigned an ET diagnosis to Case 1, despite the presence of cervical dystonia on examination. For four cases (Cases 2 – 5), assignment of dystonia diagnoses was variable, with two to three experts assigning this diagnosis, underscoring differences in diagnostic interpretation of dystonic postures on examination. SPSS (version 27) was used to compute the Cohen’s weighted Kappa statistic for each pair of raters, resulting in 21 comparisons – in 20 of 21 comparisons, diagnostic agreement was not significant (p > 0.05); only experts 4 and 5 showed significant levels of agreement (p = 0.025).

## Discussion

### Expert synopses

#### Expert 1

Although I could easily pass case 1 as ET, there are in my view several subtle signs of dystonia. In addition, the enhancement of the head tremor by vocalization and hypomimia (mild Parkinsonism) inclines me towards dystonic tremor. In case 2, the left little finger extension on pouring and walking (and also abduction), right wrist flexion in batswing position and left index finger pointing on walking incline me towards dystonic tremor. In case 3, the only dystonic feature is the flexion of the metacarpophalangeal joint of the left middle finger. Providing this does not have another cause, this would incline me towards dystonic tremor. It is an isolated sign so I would make a diagnosis of possible dystonic tremor. Otherwise the diagnosis would have been ET. In case 4, the jerky nature of this tremor, with flurries, the left index finger extension and wrist flexion with splayed and spooned fingers lead me to diagnose dystonic tremor. In case 5, the patient’s neck flexion and right index finger pointing on walking as well as left middle finger flexion at the metacarpophalangeal joint and hyperextension of the left ring finger at the distal interphalangeal joint lead me to diagnose dystonic tremor. When several of these ‘soft dystonic signs’ are present, my confidence in making a diagnosis of dystonic tremor increases.

#### Expert 2

These patients aged 72 – 88 years were diagnosed with ET. Diagnosis was based on the observation of bilateral action tremor in the upper limbs starting at 14 – 50 years of age. Signs of dystonia were not reported. The significance of mild dystonic posturing of the hands and/or fingers coexisting with tremor in all patients remains unclear since it may be also an occasional finding in otherwise healthy individuals. However, the frequency of this sign in normal people and its relationship with age are unknown. Likewise, it is unknown whether the frequency of dystonic posturing of the hands/fingers is greater in relatives of dystonic patients than in normal subjects. Information on these issues would help to advance our knowledge about the relationship between mild dystonic signs and ET. Regardless, I assigned dual diagnoses of ET and dystonia to each of these five cases.

#### Expert 3

In all cases, I assigned diagnoses of ET, but in two of them, I also diagnosed dystonia. The frequent co-existence of dystonia and ET-like tremor, and family history of both or either suggests that the two disorders share pathophysiologic mechanisms, but the nature of the relationship is still poorly understood. Dystonic tremor syndrome represents conditions where dystonia is the predominant neurological feature and tremor manifests in the body part associated with dystonia. One of the characteristic features of dystonic tremor, besides its irregularity, is “null point”. This is a position of the tremulous body part in which the tremor diminishes or resolves as the body part is allowed to assume the maximal dystonic posture [18]. Unfortunately, viewing of a video recording is not conducive to detect null point, a limitation of this study. If a patient with dystonia has a tremor in a non-dystonic body part, the tremor is described as “tremor associated with dystonia” [19]. While dystonic tremor can affect any of the body parts with dystonia, it is most frequently found in patients with cervical dystonia (as head tremor). Several early studies have demonstrated that about 25% of patients with cervical dystonia had tremor in their hands that is phenomenologically similar to ET [7]. In a more recent study of 2,362 patients enrolled in the Dystonia Coalition project, 53.3% had tremor, mostly involving the head, followed by the upper limbs and other body regions [20]. Dystonic tremor occurred in 36.9% to 48.4% of patients, but others had ET-like tremors.

#### Expert 4

Experts define ET as an isolated tremor syndrome of upper limb action tremor, designating ET plus if additional non-diagnostic neurological signs are present, including “questionable dystonic posturing,” subject to interpretation [13]. All cases display bilateral action tremors, consistent with ET. The cases demonstrate a range of subtle dystonic features. Dystonia is defined by muscle contractions causing abnormal movements or postures, often repetitive [21]. Case 1 displays mild, consistent rightward tilt of the head/neck and subtle posturing of the tremulous LUE. I assigned an additional diagnosis of dystonia largely due to the involvement of the atremulous neck. Cases 2, 4 and 5 display subtle dystonic features of tremulous limbs, not out of proportion to the tremor, consistent with underlying ET (plus). Case 3 displays subtle flexion of one finger in sustention (one position); it would be important to rule out other etiologies (e.g., musculoskeletal) and verify a recurrent pattern to confirm a dystonic component (e.g., through serial evaluation, in the clinical setting). A unifying primary dystonia appears unlikely in any of the cases based on the subtle dystonic versus prominent ET features, despite prolonged symptom duration, and the absence of dystonic features at onset. The cases highlight a common diagnostic challenge.

#### Expert 5

I assigned diagnoses of ET to each of these cases based on the severity of action tremor (i.e., moderate to severe), features of the action tremor that were typical of ET (e.g., kinetic tremor was greater than postural tremor, preferential distribution of tremor in certain joints of the upper limb [e.g., mainly wrist], regularity of oscillations, presence of a single predominant tremor axis on spirals, presence of intention tremor), longstanding duration of the action tremor (i.e., 30 or more years in each), and supporting information (e.g., presence of family history of tremor). The presence of mild dystonic postures in the limb does not preclude a diagnosis of ET, especially in the setting of longstanding and severe ET. The same rationale is applied to other disorders that are linked with the cerebellum. For example, in numerous forms of spinocerebellar ataxia, dystonic postures and movements are common [22]. Recent studies have posited a role of the cerebellum in dystonia [23]. In case 1, I also assigned diagnoses of dystonia. In that case, there was also cervical dystonia and, at present, it is not clear whether the co-occurrence of cervical dystonia merits a second diagnosis. The Jewish ancestry served to further reinforce this diagnostic choice. To be conservative, I also assigned a diagnosis of dystonia in this case.

#### Expert 6

As I know this is a study on ET with and without dystonic features, there is an inherent degree of bias in the assessment. It is evident that all patients show varying degrees, mostly very mild, of “dystonic posturing”, primarily involving the digits, especially the 5^th^ digit. However, most of these are relatively mild. Except for patient 2, where a digit is seen more prominently extending out during action, in most of the other cases, extension/flexion or splaying are seen with hands outstretched or/and in wing beating position. Logically I should have given the diagnosis of ET plus dystonia in all the cases. However, being a practical minimalist, I will stick to the ET as the primary diagnosis. All patients are age 73 – 88 years with many decades of postural/action/kinetic hand (and in some plus head) tremor, and all patients report a family history of ET or nonspecific tremor, and with no associated known secondary causes. Taken the history and examination in totality, the clinical diagnosis of ET is appropriate. For patient 5, I would like to do further clinical assessment in view of the presence of prominent jaw tremor and have raised the possibility of PD as well.

#### Expert 7

We analyzed 5 patients with young onset bilateral action and postural tremor of the upper limb, with in most a family history of ET. Age (>70) and duration of evolution (up to 57 years) were additional factors. For all, my final diagnosis based on duration >3 years, bilateral postural and action tremor, absence of rest tremor, no cerebellar ataxia and no other neurological symptoms, plus family history, was ET.

The “abnormal postures” observed were not task specific, transient, without overflow and did not get worse with action, and do not fit with the current definition of dystonia. These postures are an attempt to stabilize the hand (i) either by either co contraction of agonist and antagonist muscles to fix the joint (especially for the wrist), (ii) by increased contraction of the intrinsic muscles (with flexion of the metacarpophalangeal joints) as there is a clear contribution of the intrinsic muscles to grip and pinch strength, or (iii) by using the 5th finger as a pendulum to maintain a more steady position of the hand. The 5th finger stabilizes the hand during the grip (exclusion of the ulnar two digits resulted in a 34% to 67% decrease in grip strength) [24]. This would be a “physiological orthosis” by analogy to mechanical devices/orthosis for tremor control [25]. Therefore, these “abnormal postures” may be adaptative process in severe tremor. Overall, considering the presence of mild additional abnormal posture associated with the ET features, these patients could qualify for the diagnosis of ET “plus” according to the recent consensus classification.

### Summary of expert synopses and further discussion

This manuscript portrays some of the differences between experts in assigning diagnoses of ET or dystonia to individuals with abnormal postures on examination. For the same case, diagnoses ranged from ET (including “ET-plus”) to ET and dystonia (i.e., two diagnoses) to dystonic tremor, that is, a full spectrum of assigned diagnoses. Underlying some of this lack of agreement was a difference among some experts in terms of labeling certain postures as dystonic. Another difference lay in the interpretation of the presence of these postures in an individual who had been assigned a diagnosis of ET. To some experts, a certain severity or level of dystonia on examination was thought to be within the realm of an ET diagnosis and beyond that, a second diagnosis was warranted (i.e., dystonia). To others, the presence of any dystonia on examination was thought to require a revision of the initial ET diagnosis. Given the differences between experts, neurophysiological studies may play a role in assigning diagnoses.

The current manuscript presented five cases, carefully selected to generate discussion. The goal was not to challenge the current Consensus statement or necessarily to build consensus, but rather, to raise issues, highlight areas of uncertainty, and search for areas of common as well as differentiated thought. Several such questions are as follows:

1. Is the presence of dystonic posture on examination inconsistent with a diagnosis of ET?
2. If the presence of dystonic postures on examination is not inconsistent with a diagnosis of ET, then how much and what type of dystonic postures are allowable within the framework of an ET diagnosis?
3. Is it possible to have two simultaneous diagnoses: ET and dystonia?
4. Are some postures forms of dystonia or mere variations or normal?

Future work is needed to frame questions for future research and attempts at consensus.
